# Noninvasive Evaluation of Portal Hypertension: Emerging Tools and Techniques

**DOI:** 10.1155/2012/691089

**Published:** 2012-06-07

**Authors:** V. K. Snowdon, N. Guha, J. A. Fallowfield

**Affiliations:** ^1^MRC/Centre for Inflammation Research, QMRI, University of Edinburgh, Edinburgh EH16 4TJ, UK; ^2^Nottingham Digestive Diseases Centre, University of Nottingham, Nottingham NG7 2RD, UK

## Abstract

Portal hypertension is the main cause of complications in patients with cirrhosis. However, evaluating the development and progression of portal hypertension represents a challenge for clinicians. There has been considerable focus on the potential role of noninvasive markers of portal hypertension that could be used to stratify patients with respect to the stage of portal hypertension and to monitor disease progression or treatment response in a longitudinal manner without having to undertake repeated invasive assessment. The pathogenesis of portal hypertension is increasingly understood and emerging knowledge of the vascular processes that underpin portal hypertension has paved the way for exploring novel biomarkers of vascular injury, angiogenesis, and endothelial dysfunction. In this paper we focus on the pathogenesis of portal hypertension and potential non-invasive biomarkers with particular emphasis on serum analytes.

## 1. Clinical Importance of Portal Hypertension

**Figure 1:**
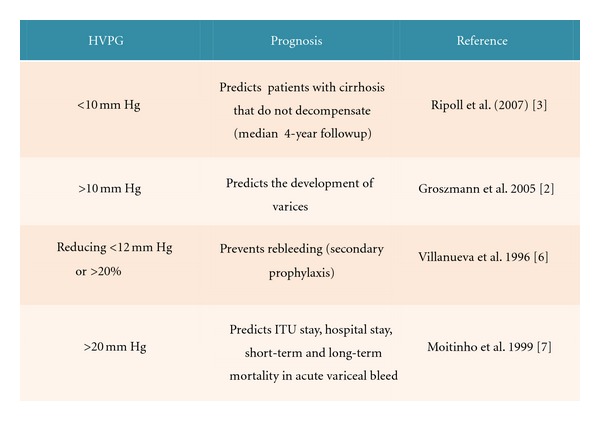
Clinical importance of portal hypertension.

Portal hypertension (PHT) is the most important consequence of cirrhosis and its presence is a hard endpoint for clinically relevant outcomes in terms of varices, ascites, hepatorenal syndrome, and encephalopathy [1]. The current gold standard for measuring PHT and its severity is measurement of the hepatic venous pressure gradient (HVPG). The prognostic value of PHT measurement at different stages in the natural history of chronic liver disease is well established, with cut-off values for the development of complications (HVPG > 10 mmHg) and variceal rupture (HVPG > 12 mmHg) [2, 3]. A reduction in HVPG (e.g., after drug therapy) below 12 mm Hg or by >20% from baseline is associated with a significant reduction in complications and death. In addition, HVPG is also emerging as a reliable endpoint to assess disease progression and therapeutic response in chronic liver disease. The importance of PHT is summarised in Figure 1 showing how changes in the HVPG affect clinical outcomes. Although HVPG measurement is safe and relatively simple to perform, it is invasive, costly, and only performed in specialist centres [4]. A recommendation from the Baverno V Consensus Workshop on Methodology of Diagnosis and Therapy in PHT was to identify noninvasive tools for detecting PHT [5], which could have clinical utility for monitoring changes in PHT over time.

## 2. Pathophysiology of Portal Hypertension

**Figure 2:**
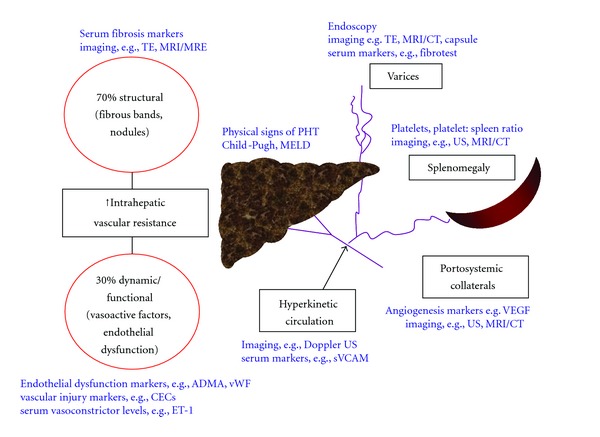
Schematic representation of the pathophysiology of portal hypertension with corresponding potential noninvasive markers. TE: transient elastography; MRI(E): magnetic resonance imaging (elastography); US: ultrasound; CECs: circulating endothelial cells; ADMA: asymmetric dimethylarginine; vWF: von Willebrand factor; ET-1: endothelin-1; MELD: Model for End-Stage Liver Disease.

In cirrhosis, PHT is initiated by an increase in intrahepatic vascular resistance (IHVR) and then exacerbated by changes in the systemic and splanchnic circulation that increase the portal inflow. Increased IHVR is caused not only by mechanical factors (e.g., fibrotic scars and regenerative nodules that distort the hepatic vascular architecture), but also by a reversible dynamic component mediated by an increase in vascular tone due to the active contraction of myofibroblasts around the hepatic sinusoids and in fibrous septa (Figure 2). This dynamic component (which accounts for ~30% of increased IHVR) reflects a functional disturbance of the liver circulation, secondary to increased production of vasoconstrictors (e.g., endothelin-1) and reduced release of endogenous vasodilators (mainly nitric oxide, NO) [6–9]. Decreased expression of endothelial NO synthase (eNOS) protein, decreased phosphorylation of eNOS by the serine-threonine kinase AKT, the presence of inhibitory substances (e.g., asymmetric dimethylarginine, ADMA), and hyporesponsiveness to NO underlie this endothelial dysfunction [10–12]. In contrast, extrahepatic endothelial cells have the opposite phenotype producing excessive NO which contributes to increased portal blood flow and an increase in PHT.

Angiogenesis has also been shown to influence PHT, with studies demonstrating that the maintenance of increased portal pressure, hyperkinetic circulation, splanchnic neovascularization, and portosystemic collateralization is regulated by vascular endothelial growth factor (VEGF) and platelet-derived growth factor (PDGF) [13].

There has been considerable interest in the identification of reliable noninvasive biomarkers for PHT including imaging techniques, routine laboratory tests, serum markers of inflammation and fibrosis, and quantitative assays of liver function which have all shown varying levels of diagnostic accuracy for PHT. The role of imaging markers has been comprehensively addressed in recent reviews [14–16]. The aim of this paper is to review the potential role of noninvasive techniques in evaluating PHT. We have focussed on serum biomarkers with particular emphasis on those that have been identified as being involved in the pathogenesis of PHT. These include novel serum markers associated with vascular injury, angiogenesis, and altered endothelial phenotypes.

## 3. Assessment of Simple Clinical Parameters

### 3.1. Clinical Manifestations of PHT

There are several clinical features that indicate the presence of PHT including ascites, splenomegaly, and caput medusa. Hypotension and tachycardia may reflect a hyperkinetic circulation. However, these signs often develop late in the natural history of PHT, can be caused by other diseases, (e.g., portal vein thrombosis or malignancy), and their presence varies between patients. A systematic review of the diagnostic accuracy of physical findings established that they had low sensitivity in compensated disease [17].

### 3.2. Platelet : Spleen Ratio (Giannini Index)

Thrombocytopaenia (platelet count <150,000/uL) is a common complication in patients with chronic liver disease. Moderate thrombocytopaenia (platelet count 50,000–75,000) occurs in ~13% of patients with cirrhosis. Multiple factors can contribute to the development of thrombocytopaenia, including splenic platelet sequestration, bone marrow suppression by chronic hepatitis C infection, and antiviral treatment with interferon-based therapy. Reductions in the level or activity of the haematopoietic growth factor thrombopoietin (TPO) may also play a role. Thrombocytopaenia has been shown to be an independent predictor of significant PHT and the presence of varices, with HVPG and platelet count showing significant negative correlation [18]. However, no specific platelet value has been found to accurately predict the presence of varices and although there is a statistical correlation, a change in the platelet count is not a reliable surrogate of reciprocal changes in portal pressure/HVPG [19]. When combined as the platelet : spleen ratio by Giannini, a 100% negative predictive value for presence of varices with a ratio of over 909 was shown [20]. This ratio has been validated and is simple and cheap [21]. However, criticisms of this simple test are that thrombocytopaenia is often a late sign of PHT, it can occur due to other conditions such as bone marrow suppression, and there is a degree of interobserver variability when measuring the spleen size.

It is worth noting that splenomegaly in cirrhosis is not simply caused by portal congestion, but is mainly due to tissue hyperplasia and fibrosis. Although a slight reduction in spleen size has been reported after liver transplantation for cirrhosis [22], evidence of regression of splenomegaly in parallel with a reduction in portal pressure is lacking. Indeed, complete resolution of splenomegaly has never been described, presumably because the architectural changes are at least in part irreversible. This calls into question the utility of splenomegaly as a dynamic marker of PHT.

### 3.3. Serum Markers of Hepatic Failure

The degree of hepatic failure as indicated by low albumin, prolonged prothrombin time, raised bilirubin, or stratification by Child-Pugh score has been shown in various studies to correlate with severe PHT and the prevalence/grade of varices. However none have been shown to correlate with the degree of PHT and are therefore not accurate enough to determine the severity of PHT in clinical practice [18, 19, 23].

## 4. Assessing the Structural Component of PHT

### 4.1. Serum Markers of Hepatic Fibrosis

The extent of hepatic fibrosis influences IHVR and therefore portal pressure, which would suggest that markers of fibrosis may also act as markers of PHT [24]. However, there have been relatively few studies exploring the use of serum fibrosis markers in PHT. Examples of potential analytes include constituents of the basal lamina (e.g., laminin) or major constituents of loose connective tissue (e.g., hyaluronic acid). These markers are found in the blood and have been correlated with hepatic fibrosis [25]. Several studies have shown that serum laminin levels correlate with HVPG in patients with fibrosis and compensated cirrhosis [26, 27]. For the prediction of severe PHT (HVPG > 12 mmHg), serum laminin had a positive predictive value (PPV) of 85% and negative predictive value (NPV) of 43% [28]. Correlation has also been shown between the serum hyaluronic acid concentration and HVPG [29]. To date, studies have only involved small numbers of patients and larger-scale studies are needed to determine the clinical utility of serum fibrosis markers for the evaluation of PHT.

FibroTest (FT) is a panel of biochemical markers that has been extensively validated for the diagnosis of advanced fibrosis and cirrhosis [14]. Thabut and coworkers conducted a prospective study in 130 patients (with or without cirrhosis) undergoing transjugular liver biopsy. The HVPG was also measured along with serum collection for FT. There was significant correlation between FT and HVPG, but this correlation was weaker in patients with established cirrhosis. The FT result was significantly higher in those with PHT, the area under the receiver operator curve (AUROC) for the diagnosis of severe PHT (HVPG > 12) was 0.79, indicating that this test was not superior to platelet count or Child-Pugh score (0.79 and 0.78, resp.) in diagnosing PHT [30]. Another study performed in 268 patients with chronic hepatitis C compared FT to other potential markers of PHT. For FT, the AUROC for the diagnosis of all varices was 0.72 and 0.76 for large varices, with a sensitivity of only 70% [16]. Despite showing promise, FT has not yet been shown to be a reliable test for clinically significant PHT.

### 4.2. Measurement of Liver Stiffness

The role of transient elastography (TE) has been explored in several reviews [14, 15]. The degree of liver stiffness has been shown to strongly predict the presence of advanced fibrosis or cirrhosis [31] and also correlates with HVPG. A very recent study by Robic and colleagues showed that the liver stiffness measurement (LSM) can be as useful as HVPG in predicting clinical decompensation and PHT-related complications [32]. In this study an LSM of 21.1 kPa or greater gave an AUROC of 0.845 for predicting portal hypertensive complications, with HVPG giving an AUROC of 0.837. No patients with an LSM <21.1 kPa developed any portal hypertensive-related complications. TE is therefore emerging as a leading diagnostic marker for PHT, although a major disadvantage of this technique is the inability to interpret scans in nearly 1 in 5 cases mostly due to obesity and limited operator experience [33]. Additionally, outside of specialist centres, many hospitals may not have access to this resource. Magnetic resonance elastography (MRE) is a promising modality for the noninvasive assessment of liver fibrosis. MRE of the spleen is also feasible and has shown promise as a quantitative method for predicting the presence of oesophageal varices in patients with advanced liver fibrosis [34]. However, MRE is currently too expensive and time consuming for widespread implementation in clinical practice.

### 4.3. Serum Markers of Angiogenesis

Both VEGF and PDGF are critical to angiogenesis, a process that contributes significantly to PHT by expanding the splanchnic vascular bed and thereby increasing portal blood flow. In addition, VEGF-dependent angiogenesis is important in portosystemic collateral vessel formation including varices. VEGF plays the predominant role in stimulating proliferation of endothelial cells and endothelial tube formation, whereas PDGF regulates vessel stability via the attachment of mural and pericyte cell populations to the endothelium. Increased VEGF expression has been shown by immunohistochemistry and western blot in the mesenteric vessels of animals with PHT, with levels correlating with increasing PHT [35]. Combined blockade of VEGF and PDGF after the development of PHT significantly decreased portal pressure and mesenteric blood flow with reduced expression of VEGF and PDGF [13]. Interestingly, this effect was not observed in models where PHT was just developing. In a model of carbon-tetrachloride- (CCl_4_-) induced cirrhosis, animals with PHT had significantly increased levels of intestinal and plasma VEGF but there was no correlation between plasma VEGF levels and portal pressure [36]. This contrasts with a small human study investigating the role of Octreotide in PHT which showed a significant correlation between HVPG and the serum VEGF level [37]. It appears that VEGF and PDGF have a synergistic interaction in the pathogenesis of PHT through regulation of splanchnic neovascularisation and portosystemic collateral formation. However, data to support a diagnostic role for these markers in PHT is currently lacking.

In patients with cirrhosis, serum levels of soluble vascular adhesion molecule (sVCAM-1) have been associated with increasing liver fibrosis and are related to angiogenesis. Although serum sVCAM-1 levels did not correlate with HVPG, it could represent a marker of the hyperkinetic circulation and levels were closely related to clinical stage (Child-Pugh, MELD scores) [38].

## 5. Dynamic Functional Component of Portal Hypertension

### 5.1. Markers of Increased Vasoconstriction

As PHT is associated with hyperproduction of endogenous vasoconstrictors, measurement of these factors in the serum could be used to evaluate PHT noninvasively. Serum endothelin-1 (ET-1) levels are elevated in portopulmonary hypertension and associated with a poor outcome [39] and have also been shown to correlate with HVPG values in patients with cirrhosis [40]. Thus, serum endothelin levels could be used to evaluate the degree of PHT, although further studies are needed to determine the clinically relevant levels.

Urotensin II (U-II), a somatostatin-like cyclic peptide, was recently identified as the most potent human vasoconstrictor peptide. One study suggested that U-II was an important marker of the severity of PHT in children with chronic liver disease and correlated with Child-Pugh score, paediatric end-stage liver disease score, and long-term clinical outcome [41]. In another study, in adults with cirrhosis and hyperkinetic circulation but with normal serum creatinine, U-II levels were notably higher than in healthy subjects; however there was no correlation with cardiac index or other haemodynamic parameters observed [42].

### 5.2. Markers of Endothelial Dysfunction

Endothelial dysfunction is a major determinant of the increased intrahepatic vascular tone observed in cirrhosis and a number of markers reflecting this dysfunction have been identified.

NO synthesis can be inhibited by the endogenous circulating amino acid asymmetric dimethylarginine (ADMA). ADMA is synthesized via enzymatic methylation of L-arginine residues in proteins and is released during proteolysis and metabolized to citrulline and dimethylamine in the liver, with impaired liver function associated with increased plasma levels of ADMA. There have been several studies linking ADMA to endothelial dysfunction in cardiovascular disease and multiorgan failure [43, 44]. Laleman and colleagues examined different animal models of cirrhosis and PHT and showed that bile-duct-ligated (BDL) animals exhibited normal eNOS levels in contrast to thioacetamide and carbon-tetrachloride-induced models of cirrhosis, suggesting that posttranslational regulatory mechanisms are involved in the defective production of NO in some causes of cirrhosis [12]. In BDL-treated animals ADMA levels were significantly elevated suggesting a possible role for ADMA in inhibiting eNOS. Lluch and coworkers showed that peripheral blood levels of ADMA correlated with the degree of liver failure and decompensation in patients with alcohol-related cirrhosis [45]. In a further study involving patients with compensated chronic hepatitis C cirrhosis, a positive statistically significant correlation was found between HVPG and ADMA [46]. This was the first study to observe a correlation between the degree of PHT and ADMA levels. Further mechanistic studies are needed to define ADMA metabolism and function in PHT.

Von Willebrand factor (vWF), P-selectin, and 8-iso-PGF2a have also been identified as surrogate markers of endothelial dysfunction and levels of these factors are increased in patients with cirrhosis compared with controls. In patients with PHT, vWF levels significantly correlated with HVPG, Child-Pugh, and MELD scores. In addition, peripheral vWF levels with a cut-off value of 216 U/dL (Youden index) were also predictive of clinical outcomes (PHT-related events and liver transplantation) [47].

### 5.3. Markers of Vascular Injury: Circulating Endothelial Cells (CECs)

CECs are a specific population of endothelial cells in peripheral blood. They exceed 10 *μ*m in size and are characterized by the expression of at least two different endothelial markers and absence of expression of leukocyte markers [48]. They are present in very low levels in healthy individuals. Elevated levels of CECs have been observed in a variety of diseases associated with vascular damage and are considered to reflect the severity of vascular injury [11]. Abdelmoneim and colleagues [49] performed a small study on patients with cirrhosis, with or without PHT, the latter being defined by the presence of varices, splenomegaly, ascites, encephalopathy, and/or HCC versus age- and sex-matched controls. The number of CECs was significantly elevated in patients with cirrhosis compared to controls. However, HVPG was not measured in these subjects such that conclusions regarding the clinical potential of CECs as a biomarker for PHT are limited. When combined with the platelet count (PC) as CEC/PC with a cut-off value of 0.21, the sensitivity for diagnosing cirrhosis was 100% with a specificity of 73% and AUROC of 0.8. Additionally correlation was seen with a rising CEC/PC and presence of decompensation. A further larger study is needed in patients where CECs levels and CEC/PC are correlated with the HVPG.

## 6. Markers of Modifications in Splanchnic Circulation and Hyperkinetic Syndrome

The extrahepatic endothelial phenotype is that of excess NO production causing peripheral vasodilatation and increased blood flow through the mesenteric vessels and portal vein. This exacerbates the portal pressure. Imaging of the portal and systemic circulation has been performed using duplex Doppler ultrasound, CT, and MRI. Detailed discussion of these modalities is beyond the scope of this paper but noninvasive imaging has shown promise in detecting portosystemic collaterals and changes in portal vein expiration diameter, hepatic vein waveforms, and splenic pulsatility which all have varying discriminatory ability in detecting changes in PHT [14].

## 7. Video Capsule Endoscopy (VCE)

The presence of varices is objective evidence of the presence of severe PHT. Rather than pure search for surrogate markers of PHT, there has been much interest in the use of capsule endoscopy in diagnosing varices. Promising results in pilot studies led to two larger studies. De Franchis et al. [50] showed, in a study of 288 cirrhotic patients undergoing endoscopy for either screening or surveillance, that VCE had 84% sensitivity and 92% PPV for detecting all oesophageal varices. For determining the size of the varices and need for surveillance versus treatment, it was shown that VCE had an 87% PPV and 92% NPV suggesting that as a noninvasive tool it is promising [50]. Lapalus et al. [51] showed similar encouraging results in a study of 120 patients with PHT undergoing VCE followed by endoscopy. They found VCE had 77% sensitivity and 90% PPV for diagnosing oesophageal varices [51]. Concordance between the two blinded endoscopists was good, particularly with regard to who required prophylaxis. However, recent evidence from a clinical study by Chavalitdhamrong et al. [52] has shown overall accuracy for detection of oesophageal varices at only 63.2% with 51.5% sensitivity for other significant upper GI lesions such as portal hypertensive gastropathy or gastric varices, suggesting that there are fairly major discrepancies in the sensitivity and specificity between operators [52]. It is clear that standard endoscopy is superior to VCE. Although it does show promise as a noninvasive tool, its role may be in patients who require screening whilst on treatment, but do not tolerate standard endoscopy well.

## 8. Conclusion

PHT is a robust outcome measure which has proven prognostic significance in chronic liver disease and the potential for use in monitoring disease progression and treatment efficacy. In this paper we have outlined the pathogenesis of PHT and discussed a range of candidate serum biomarkers that have been identified. At present, transient elastography appears to represent the most promising noninvasive technique that could potentially replace HVPG measurement for PHT or endoscopy for variceal detection. The potential role of serum markers for the evaluation of PHT remains unproven, but will increasingly be assessed in prospective clinical studies. Further advances in our understanding of the underlying mechanisms responsible for the development and progression of PHT will continue to reveal additional biomarker targets.

## 9. Methods

Referred papers were identified by MEDLINE search through the PubMed database by combining the keyword “portal hypertension” with the keywords “biomarkers, serum, fibrosis, endothelial cell and angiogenesis.” Additional papers were identified by searching of references through retrieved papers.
